# Concrete Waste and CDW Powders as Portland Cement Replacement in Mortar: A Preliminary Study

**DOI:** 10.3390/ma19030519

**Published:** 2026-01-28

**Authors:** Daniel Suarez-Riera, Giuseppe Ferrara, Luca Lavagna, Devid Falliano, Matteo Pavese, Luciana Restuccia, Jean-Marc Tulliani

**Affiliations:** 1 Department of Structural, Building and Geotechnical Engineering, Politecnico di Torino, C.so Duca Degli Abruzzi 24, 10129 Torino, Italy; daniel.suarez@polito.it (D.S.-R.); devid.falliano@polito.it (D.F.); luciana.restuccia@polito.it (L.R.); 2 Department of Applied Science and Technology, Politecnico di Torino, C.so Duca Degli Abruzzi 24, 10129 Torino, Italy; giuseppe.ferrrara@polito.it (G.F.); matteo.pavese@polito.it (M.P.); jeanmarc.tulliani@polito.it (J.-M.T.)

**Keywords:** concrete waste powder, construction and demolition waste, cement replacement, sustainability, circular economy

## Abstract

The construction industry’s heavy reliance on Ordinary Portland Cement (OPC) significantly contributes to global CO_2_ emissions, prompting the search for sustainable alternatives. This study investigates the partial substitution of Portland cement with construction and demolition waste (CDW) powder and concrete waste (CON) powder in mortar mixes. Replacement levels of 5%, 10%, 15%, and 20% by weight were tested following EN 196-1 standards to evaluate the mechanical performance of the resulting materials. X-ray diffraction (XRD), X-ray fluorescence (XRF), and thermo-gravimetric analyses confirmed that CDW and CON powders consist mainly of quartz and calcite, with chemical compositions compatible with cementitious systems. Mechanical testing revealed that compressive strength was maintained or slightly improved at replacement levels up to 10%, while higher substitutions led to moderate reductions due to dilution effects. The use of CDW and CON powders effectively transformed a 52.5 R Type I cement into a 42.5 R Type II equivalent, demonstrating the feasibility of producing sustainable binders with acceptable performance.

## 1. Introduction

Ordinary Portland Cement (OPC), the primary binder in concrete and mortar, is a cornerstone of modern construction due to its versatility, strength, and durability. Nevertheless, urbanization and industrialization have substantially increased its global production over the past few decades [1]. In 2023 alone, global cement production was estimated to be around 4.03 billion metric tons [2]. This staggering volume underscores its critical role in construction and highlights significant environmental concerns. OPC’s production is energy-intensive and a major contributor to global CO_2_ emissions. It is estimated that cement production accounts for approximately 8% of the world’s anthropogenic CO_2_ emissions [3,4].

The environmental footprint of OPC production necessitates a shift towards more sustainable practices. Furthermore, the Global Cement and Concrete Association has set ambitious goals, including a 25% reduction in CO_2_ emissions by 2030 and full decarbonization by 2050 [5]. This includes improving energy efficiency, adopting alternative fuels, and, crucially, incorporating supplementary cementitious materials (SCMs) to partially replace Portland cement in concrete mixtures [6,7,8].

Natural alternative binders or industrial by-products with hydraulic and/or pozzolanic behavior can be adopted in cement blends to reduce clinker use. The most commonly adopted supplementary cementitious materials (SCMs) are limestone, ground-granulated blast furnace slag (GGBS), and coal fly ash [9]. Other products adopted in minor amounts are silica fume, natural pozzolans, calcined clay, and other amorphous or imperfectly crystalline materials containing silica, alumina, and/or lime [10].

The utilization of alternative materials in place of Portland cement not only mitigates the environmental impact but also enhances certain properties of concrete. Waste products, such as construction and demolition waste (CDW) and concrete waste powder, present a promising avenue for sustainable development. When processed and incorporated into cementitious mixtures, these materials can reduce the reliance on virgin OPC, thus lowering the overall CO_2_ emissions [11].

CDW constitutes a substantial portion of global waste generation, varying in quantity and composition depending on factors such as population density, construction activity, material usage, and regional practices. According to the 2018 fact sheet by the Environmental Protection Agency (EPA), demolition activities account for approximately 90% of total CDW, while only 10% originates from new construction [12]. Additionally, in the United States alone, the construction sector generated approximately 600 million tons of CDW in the same year, which is more than twice the amount of municipal solid waste produced [13]. Despite the significant environmental impact of CDW, many of its components, including concrete and metal, can be effectively recycled, whereas materials such as bricks, clay tiles, and gypsum drywall present greater challenges to recycling [14]. In addition, in the European Union, approximately 3 billion tons of waste are produced annually, with construction and demolition activities accounting for about one-third of this total [15,16]. The average recovery rate is close to 50%, though it varies considerably across member states, ranging from as low as 10% to as high as 80%. For example, Italy achieves a recovery rate of nearly 80%, while France stands at 48%, Spain at approximately 40%, and Germany at 34% [17]. In the specific context of Turin, where this study is focused, the annual generation of CDW was estimated at 4.3 million tons in 2013. The current recycling rate is around 50%, with most of the resulting recycled aggregates being utilized for environmental backfilling or road construction.

The pressing need to mitigate the environmental burden of CDW has prompted the construction industry to explore sustainable solutions, such as the valorization of CDW materials, to reduce the exploitation of virgin resources and minimize landfill disposal. Among CDW materials, concrete represents the most rapidly increasing recyclable component, accounting for 31.5% of the CDW market in 2020, with growing applications in civil engineering.

While multiple studies have already investigated the structural behavior of recycled aggregates (RAs) and recycled concrete aggregates (RACs), variations in research outcomes highlight the necessity for further exploration to optimize the utilization of CDW. Beyond its role as a source of recycled aggregates, CDW represents a significant potential source of supplementary cementitious materials (SCMs). In fact, various CDW components can be crushed and ground into fine powders suitable for partial cement replacement. For instance, concrete waste powder derived from the recycling of old concrete structures has been successfully used to replace a fraction of OPC, reducing the demand for virgin raw materials and lowering carbon emissions associated with cement production [18,19,20]. These waste materials, which would otherwise be landfilled, can be repurposed to develop more sustainable cement-based materials, aligning with circular economy principles.

Thus, this study aims to assess the impact of partially replacing Portland cement with CDW and concrete waste powder at varying levels (5%, 10%, 15%, and 20%) to determine the optimal substitution rates that preserve or enhance the mechanical performance of the resulting mortar. By emphasizing the need for further investigation into the microstructural and mechanical behavior of CDW-based cement replacements, this research highlights the importance of developing standardized guidelines to facilitate their practical implementation in sustainable construction. This study uniquely explores the partial replacement of Portland cement with construction and demolition waste (CDW) and concrete waste (CON) powders, demonstrating their potential to reduce the environmental impact of cement production. The findings highlight the feasibility of transforming a 52.5 R Type I cement into a more sustainable 42.5 R Type II equivalent, emphasizing the practical applications of waste materials in the construction industry.

## 2. Materials and Methods

Portland cement Type I, 52.5 R (Buzzi-Unicem, Casale Monferrato, Italy) (Table 1), CEN Standard sand (Société Nouvelle du Littoral, Leucate, France), tap water, construction and demolition waste powder (CDW), and concrete waste powder (CON) recovered from construction and demolition waste activities in Torino, Italy, were the materials used for this experimental study.

The industrial supplier of the recycled aggregate did not indicate a specific pretreatment for the materials used in this work, but the main processes are typically the following:

- The first step in processing is crushing, which involves using a crusher to break down the waste material into smaller pieces. The crusher used in this process can be either primary or secondary. The primary crusher is used to break down larger pieces of waste material, while the secondary crusher is used to reduce the size of the crushed material further;
- After the crushing process, the material is then screened to remove any contaminants that may be present. The screening process involves passing the crushed material through a series of screens with different mesh sizes. The screens are designed to separate the material into different sizes, with the larger pieces being returned to the crusher for further processing.
- Once the material has been screened, it is then sorted to remove any non-aggregate materials, such as plastics and wood. This process is critical to ensure the resulting material is high-quality and suitable for construction projects. However, since the sorting process can be carried out manually or using automated equipment, not all unwanted particles are expected to be removed.
- The final step in the processing of recycled aggregates is washing. This process involves the use of water to remove any remaining contaminants that may be present in the material. The washed material is then dried to remove any excess moisture. The quantity of water absorption is around 5%wt. The CDW/CON powders used in this research were the passing fraction of a sieving process to obtain bigger fractions used in another study [27].

Five mortar specimen series were made, with different substitution percentages by weight of cement with CDW and CON (0%, 5%, 10%, 15%, and 20%). These percentages were chosen to try to mimic the amount of limestone that is used in Type II/A cements (UNI EN 197-1 [28]). The samples were prepared in adherence to the EN 196-1:2016 standard [26], with a water-to-cement ratio of 0.5 and a cement-to-aggregate ratio of 1:3, as outlined in Table 2. Each series comprised a minimum of three samples. The mixing procedure involved adding water to cement in a bowl, stirring at low speed for 30 s, gradually introducing sand over the next 30 s while increasing to high speed, and pausing for 90 s to scrape off any mortar adhering to the bowl’s walls. After a brief 15 s rest, mixing resumed at high speed for another 60 s. The prepared mixture was then poured into steel molds, yielding three 40 × 40 × 160 mm^3^ prismatic specimens. The molds were first half-filled and compacted with 60 jolts, then the remaining mixture was similarly poured and compacted with another 60 jolts. The molds were subsequently stored in a climate-controlled chamber for 24 h at room temperature (RT, 24 ± 1 °C) and 95% relative humidity. After this period, all specimens were transferred to a water tank and maintained at RT for additional curing periods of 7 and 28 days. After curing, the samples underwent three-point bending and compression tests to assess their mechanical performance, adhering to the EN 196-1 standard.

### 2.1. CON and CDW Characterization

X-ray diffraction (XRD) patterns were obtained using the X-ray diffractometer PW3040/60 X’Pert PRO MPD from PANalytical, Almeno, The Netherlands in a Bragg–Brentano geometry, with Cu Kα anode source at 40 kV and 40 mA (*λ* = 0.154056 nm). The chemical composition was assessed by X-Ray Fluorescence (XRF) using Rigaku ZSX 100E (Supermini 200, Tokyo, Japan). The test was carried out by adopting a semiquantitative analysis calculated by a Fundamental Parameters (FP) program using a built-in sensitivity library. Thermo-gravimetric analysis was conducted in air in a TG-DTA instrument LABSYS EVO from Setaram, Caluire-et-Cuire, France. Samples were heated from 25 °C to 1000 °C with a constant heating ramp of 10 °C min^−1^. The air was supplied with a constant flow rate (50 mL min^−1^). The particle size distribution was assessed using a Mastersizer 3000+ Ultra laser granulometer, Worcestershire, United Kingdom.

### 2.2. Mechanical Characterization

The mechanical performance of mortar specimens was assessed through three-point bending (TPB) and compression tests following the UNI 196-1 Standard. The TPB test was carried out using a Zwick-Line Z050 single-column machine, Genova, Italy with a cell load capacity of 50 kN, a pre-load of 5 N, a span of 100 mm, and a testing rate of 50 N/s. The flexural strength was determined using the following equation:

(1) $$\sigma_{f}=\frac{3F_{max}L}{2bh^{2}}\;\;\;[MPa]$$

in which: *F_max_* is the maximum applied force on the prism at the instant of failure [N], *L* is the effective span [mm], *b* is the prism width [mm], and *h* is considered the height of the specimen under the point of the application of the load [mm].

Following the flexural testing, the fractured sections of the prisms underwent compression testing utilizing a Zwick–Baldwin single-column apparatus, Genova, Italy equipped with a load cell capable of handling 500 kN and operating at a test velocity of 2400 N/s. The compressive strength (σ_c_) was calculated by dividing the maximum load by the initial cross-sectional area of the specimen:

(2) $$\sigma_{c,max}=\frac{F_{max}}{bh}\;\;\;[MPa]$$

## 3. Results and Discussion

**Figure 1:**
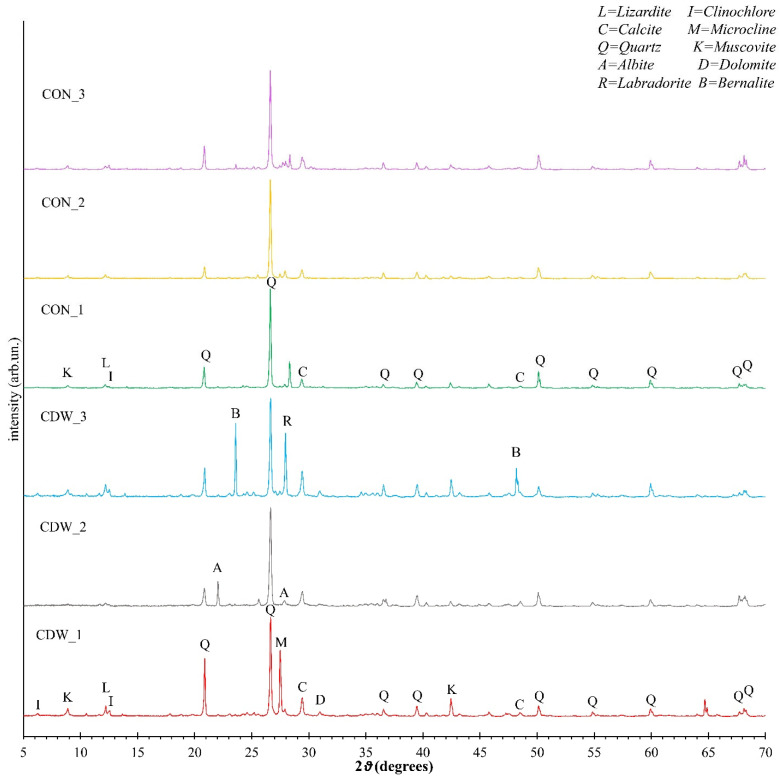
XRD results.

XRD analyses of CDW and CON are shown in Figure 1. The analyses show patterns that are somewhat similar, regardless of the retained fraction. They revealed the presence in all the fractions of quartz (SiO_2_) and calcite (CaCO_3_) as major constituents, with main peaks, respectively, at 26.6° and 29.4°. At the same time, clinochlore (magnesium iron aluminum aluminosilicate, (Mg,Fe)_6_(Si,Al)_4_O_10_(OH)_8_), muscovite (hydrated phyllosilicate mineral of aluminum and potassium, KAl_3_Si_3_O_10_(OH)_2_), albite (sodium aluminosilicate, NaAlSi_3_O_8_), lizardite (an aluminosilicate mineral belonging to the serpentine group, (Mg,Al)_3_[(Si,Fe)_2_O_6_](OH)_4_), cordierite (magnesium iron aluminum cyclosilicate, Mg_2_Al_4_Si_5_O_18_), dolomite (calcium magnesium carbonate, (Ca,Mg)CO_3_), microcline (potassium aluminosilicate, KAlSi_3_O_8_), labradorite (sodium calcium aluminosilicate (Ca,Na)(Al,Si)_4_O_8_), and bernalite (iron hydroxide, Fe(OH)_3_) were recognized as secondary phases. The attribution of some of these minority phases is uncertain. The peaks of the various phases are indicated in Figure 1 with a letter, as shown in the legend.

Moreover, it must be stressed that X-ray diffraction cannot observe amorphous or poor crystalline phases. Gypsum was never found in the samples investigated. These phases, i.e., clinochlore, albite, lizardite, quartz, and dolomite, come from the aggregate fraction, while calcite could have different origins: from aggregates, as a cement filler, and from the concrete degradation process (carbonation). Overall, the slight variations observed in all the tests taken on different portions of CDW or CON could potentially be attributed to the lack of sample homogeneity.

Table 3 shows the composition of CDW and CON powder from XRF analysis. The high presence of silica (43.7% and 42.3%, respectively) is due to the presence of silicates and quartz. Calcium, magnesium, aluminum, and iron are consistent with the typical cement composition. The presence of quartz and calcite is also confirmed by intense peaks in the XRD pattern (Figure 1).

**Figure 2:**
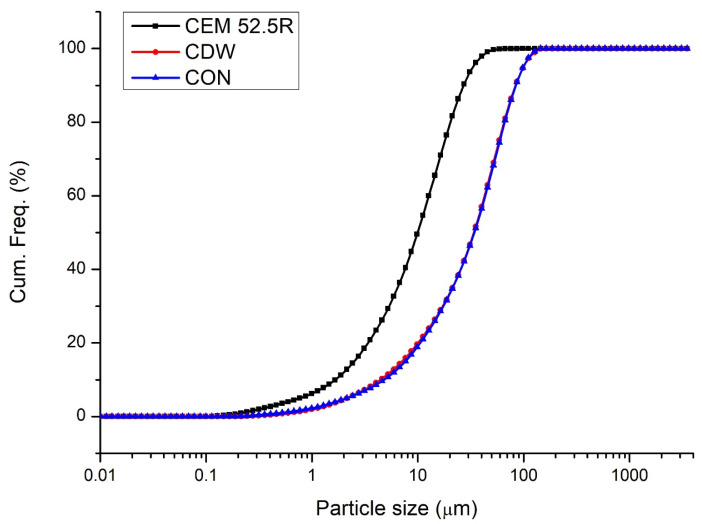
Particle size distribution of cement (black), CDW (red), and CON (blue) powder.

The particle size distribution curves (Figure 2) highlight clear differences between cement (CEM 52.5 R) and the two recycled powders (CDW and CON) used as clinker substitutes. The D10, D50, and D90 for cement are, respectively: 1.88 μm, 11.3 μm, and 30.7 μm; for CDW are, respectively: 4.43 μm, 33.9 μm, and 89.9 μm; for CON are, respectively: 4.80 μm, 34.2 μm, and 84.3 μm.

The cement exhibits a markedly finer granulometry, with its cumulative curve shifted to the left relative to the recycled materials. This indicates that a substantial portion of cement particles lies in the sub-10 µm range, which is characteristic of Portland cement and directly associated with high early reactivity due to the increased specific surface area.

Conversely, both CDW and CON display very similar granulometric profiles, almost overlapping across the entire size range. Their median particle size (D50) is significantly larger than that of cement, with the main fraction lying between approximately 10 µm and 100 µm. This suggests that—despite fine grinding—the recycled powders retain a coarser texture compared to clinker, likely due to the presence of unreacted aggregates, old hydration products, and residual micro-aggregates typical of demolition-derived materials.

The coarser recycled powders behave predominantly as filler materials, contributing mainly to particle packing rather than chemical reactivity.

Overall, the graph highlights why substitution levels of recycled powders must remain controlled: their significantly coarser granulometry compared to cement inherently limits their reactivity. However, the similar particle size distributions of CDW and CON also indicate good process reproducibility, supporting their potential use as consistent clinker replacement materials when properly processed.

**Figure 3:**
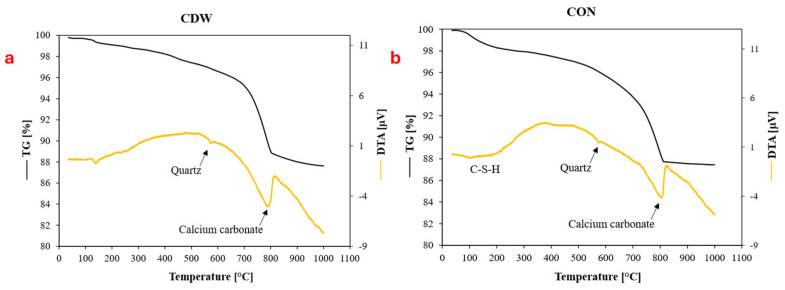
Thermo-gravimetric analysis of CdW (**a**) and CON (**b**) under air atmosphere.

The TG-DTA curves of CDW and CON powder samples are illustrated in Figure 3. For the CDW sample (Figure 3a), an endothermal peak around 140 °C indicates the dehydration of ettringite, confirmed by a corresponding mass loss in the TG curve [27]. At higher temperatures, the mass loss corresponds to the progressive dehydration of C-S-H. For the CON sample (Figure 3b), the mass loss up to around 600 °C is attributed to the progressive dehydration of C-S-H [29]. In both the CDW and CON samples, the DTA signal at 573 °C represents the α-to-β quartz transition, likely due to the presence of aggregates in the powder. The endothermic peak between 600 and 700 °C and 800 °C corresponds to the decomposition of more or less crystallized calcium carbonate, which constitutes almost 20% of the samples [27]. The presence of quartz and calcium carbonate aligns with the results from XRD analysis.

**Figure 4:**
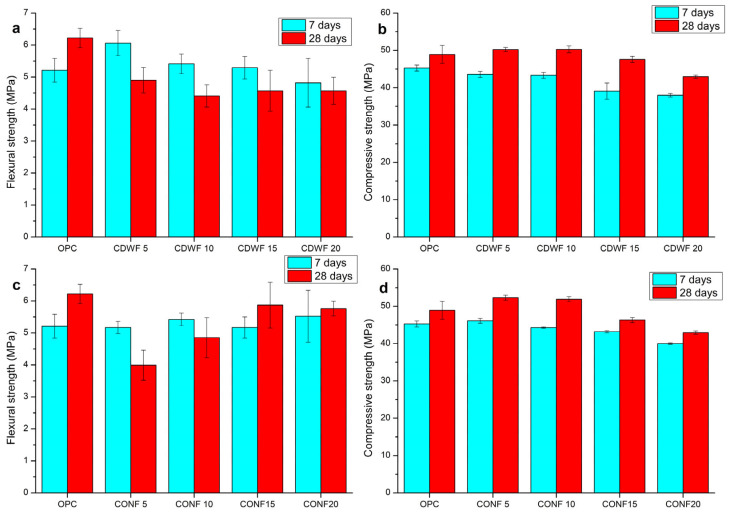
Flexural (**a**,**b**) and compressive strength of CDW series; (**c**) flexural and (**d**) compressive strength of CON series.

Regarding the mechanical performance of the mortar, Figure 4 illustrates the flexural and compressive strength characteristics of mortars incorporating CDW-F and CON-F at 7 and 28 days. For flexural strength, the figure indicates that as the proportion of the fine fraction increases (5%, 10%, 15%, and 20%), the bending capacity generally decreases at 28 days. Specifically, the CON-F 5 and CDW-F 10 samples exhibited significant reductions of 36% and nearly 30%, respectively, compared to the control group (OPC). However, the flexural strengths of the CDW-F 15 and CON-F 20 samples remained relatively close to the reference.

Regarding compressive strength (Figure 4b,d), the results demonstrate promising performance up to a 10% substitution level, with a 5% improvement over the reference mortar at 28 days. This improvement can be attributed to the absorption capacity of the CDW and CON used to replace the cement, which absorbs the free water present in the mix, thereby affecting the water-to-cement ratio and resulting in higher compressive strength values. Beyond a 10% replacement level, this effect is diminished as cement is replaced to a greater extent, and the CDW-F and CON-F do not react with water, acting merely as filler materials.

The very similar mechanical behavior observed for CON and CDW powders can be directly correlated with their comparable chemical and physical characteristics. As shown by XRF analysis (Table 3), both materials exhibit closely aligned oxide compositions, dominated by SiO_2_ and CaO (in the form of CaCO_3_), with similar contents of Al_2_O_3_, Fe_2_O_3_ and MgO, which are typical of cementitious and aggregate-derived phases. This compositional similarity is further confirmed by XRD results, where both powders mainly consist of quartz and calcite, with minor aluminosilicate phases. All these phases are not very reactive, suggesting very limited, if present, pozzolanic activity.

In addition, the particle size distribution of CON and CDW powders is almost overlapping (Figure 2), with comparable D10, D50, and D90 values. The coarser granulometry than Portland cement limits their possible intrinsic reactivity and promotes a predominantly filler-like behavior in the cement matrix. As a consequence, both CON and CDW powders influence the hydration and mechanical performance of mortars in a similar manner, mainly through dilution and particle packing effects rather than through significant pozzolanic activity.

Table 4 provides a comparative overview of recent studies investigating the partial replacement of clinker with fine recycled materials derived from concrete demolition (RCF, RCP, RFP, and CDW powders). Overall, the findings show that cement substitution with recycled fines is technically feasible, especially at low to moderate replacement levels (≤20%), but performance strongly depends on the processing and activation of the recycled material.

In most cases, recycled concrete fines exhibit a coarser particle size distribution compared to Portland cement, as confirmed by the granulometric curves presented in Figure 2. While cement particles are predominantly concentrated in the sub-10 µm range, recycled powders typically show median particle sizes (D50) in the range of 20–60 µm. This granulometric mismatch, together with the phase composition of the particles, explains the generally low intrinsic reactivity of CDW-derived powders, which mainly act as inert or weakly reactive fillers rather than true cementitious phases. Consequently, at replacement levels exceeding 20–25%, most studies report a progressive reduction in compressive and flexural strength due to dilution of clinker content and reduced availability of early hydration products.

Nevertheless, the literature (Table 4) indicates that low to moderate replacement levels (≤15–20%) are often compatible with acceptable mechanical performance. At these substitution ratios, the recycled powders can contribute positively through physical effects such as particle packing optimization and nucleation of hydration products, partially compensating for their limited chemical activity. This trend is consistently observed across studies using mechanically ground RCF, RCP, and fine CDW powders.

More advanced processing techniques significantly enhance the performance of recycled powders. Mechanical activation, achieved through high-energy milling, increases the specific surface area and induces structural disorder in the hydrated and unhydrated phases, leading to measurable improvements in strength development. Thermal activation and carbonation treatments further modify the mineralogical composition of recycled powders, promoting the formation of reactive calcium-rich phases or stable carbonates that enhance interfacial bonding. In several studies, these treatments enabled clinker replacement levels up to 20–30% while maintaining mechanical properties comparable to reference cement pastes and mortars. However, such activation methods entail additional energy consumption and processing costs, which must be carefully evaluated in life-cycle assessments.

The quality and origin of CDW also play a critical role. Fine fractions obtained from high-quality parent concrete exhibit superior performance compared to mixed or contaminated demolition waste, emphasizing the importance of selective demolition, sorting, and beneficiation processes. Studies focusing on fine demolition recyclates (0–2 mm) report large variability in mechanical results, reinforcing the need for standardized processing routes to ensure consistent material properties.

Overall, the literature reviewed in Table 3 confirms that recycled concrete and CDW fine powders represent a technically viable pathway for clinker reduction, particularly when used at controlled replacement levels and combined with appropriate activation strategies. The main challenge remains balancing mechanical performance, processing energy, and environmental benefits to achieve truly sustainable low-carbon cement formulations.

## 4. Conclusions

Incorporating CDW and concrete waste powder into cement paste and mortar aligns with the principles of the circular economy, promoting resource efficiency and waste minimization. The construction industry can significantly reduce its environmental impact while maintaining structural integrity and performance by substituting a fraction of Portland cement with these alternative materials. Consequently, the decision was made to employ this fraction as a partial replacement for Portland Cement at four distinct percentages: 5%, 10%, 15%, and 20%. This substitution transformed the 52.5 R cement from Type I to a composition resembling 42.5 R Type II cement.

From an application perspective, the results indicate that both CDW and CON powders can be effectively used as low-impact clinker replacement materials at substitution levels up to 10%, allowing the production of mortars with mechanical performance comparable to conventional OPC systems. This approach is particularly suitable for non-structural and semi-structural applications, such as masonry mortars, rendering, screeds, and general-purpose concretes, where the use of 42.5 R Type II cement is commonly required.

Future research should focus on improving the reactivity of CDW and CON powders through targeted activation strategies, such as finer grinding, thermal or chemical activation, and controlled carbonation. Moreover, durability-related aspects, including long-term strength development, shrinkage, permeability, and resistance to aggressive environments, should be systematically investigated to support the large-scale implementation of these recycled powders in sustainable cementitious systems.

The shift towards sustainable construction practices is imperative for mitigating the environmental impact of Portland cement production. Integrating waste products like CDW and CON waste powder into cementitious materials offers a viable pathway to achieve this goal. Ongoing research and development in this field are crucial to optimizing the use of these alternative materials, ensuring they meet the required performance standards while significantly reducing the construction industry’s carbon footprint.

## Figures and Tables

**Table 1:** Physical and chemical characteristics of the cement from the supplier.

Parameter	Test Method	Indicative Value	Characteristic Limits Based on the Standard/Norm
Sulfate (SO_3_)	UNI EN 196/2 [21]	<3.7%	≤4.0%
Chlorides (Cl^−^)	UNI EN 196/2	<0.08%	≤0.10%
Loss of fire	UNI EN 196/2	<5.0%	≤5.0%
Insoluble residue	UNI EN 196/2	<1.0%	≤5.0%
Chromium VI soluble in water	UNI EN 196/10 [22]	≤2 ppm	≤2 ppm
Blaine specific surface area	UNI EN 196/6 [23]	4000–5500 cm^2^/g	
Setting starting time	UNI EN 196/3 [24]	>90 min	≥45 min
Volume stability	UNI EN 196/3	≤10 mm	≤10 mm
Mortar consistency	UNI 7044 [25]	>70%	
Compression resistance after 2 days curing	UNI EN 196/1 [26]	>35.0 MPa	≥30.0 MPa
Compression resistance after 28 days curing		>56.0 MPa	≥52.5 MPa

**Table 2:** Mix design.

	Specimen ID	w/c	Cement (g)	CDW/CON (g)	Water (g)	Standard Sand (g)
Mortar samples	OPC	0.5	450	-	225	1350
	CDW-F 5	0.5	427.5	22.5	225	1350
	CDW-F 10	0.5	405.0	45.0	225	1350
	CDW-F 15	0.5	382.5	67.5	225	1350
	CDW-F 20	0.5	360.0	90.0	225	1350
	CON-F 5	0.5	427.5	22.5	225	1350
	CON-F 10	0.5	405.0	45.0	225	1350
	CON-F 15	0.5	382.5	67.5	225	1350
	CON-F 20	0.5	360.0	90.0	225	1350

**Table 3:** XRF Results for CDW and CON.

Component	Result (Mass %)	
	CDW	CON
LOI-Flux	12.10	12.00
Na_2_O	1.18	1.38
MgO	6.18	4.75
Al_2_O_3_	12.40	9.10
SiO_2_	43.70	42.80
SO_3_	2.26	3.42
Cl	0.14	0.079
K_2_O	1.96	1.73
CaO	13.50	19.30
TiO_2_	0.54	0.39
Cr_2_O_3_	0.071	0.067
MnO	0.17	0.17
Fe_2_O_3_	5.42	4.49
P_2_O_5_	0.23	0.16
NiO	0.038	0.033
CuO	0.011	0.011
ZnO	0.022	0.017
As_2_O_3_	0.010	0.009
Rb_2_O	0.011	0.010
SrO	0.032	0.037
Y_2_O_3_	0.010	0.002
ZrO_2_	0.028	0.035

**Table 4:** Comparison with literature.

Material	Replacement Level	Mechanical Results	Key Notes	Reference
Recycled concrete fines (RCF/RCP)	5–30%	Small loss ≤20% replacement; large drop ≥30%	Finer grinding improves reactivity	[30]
Recycled concrete powder (RCP)	5–25%	Strength generally decreases with replacement	Performance depends on PSD and carbonation state	[31]
Recycled concrete fine powder (RFP)	5–20%	Small reductions ≤15%; optimal ≤20%	Acts as filler; finer grinding helps	[32]
Thermally activated recycled powder	10–30%	Activation recovers strength; 20% ≈ control	Thermal + mechanical activation increases reactivity	[33]
Carbonated recycled powder (CRP)	Up to 20%	Carbonation improves flexural +27.8%, compressive +20%	CO_2_ carbonation improves early/late strengths	[34]
Recycled powder from CDW	5–30%	≤20% feasible with acceptable strength losses	Activation and PSD control essential	[35]
Mechanically activated fines (MA-RCF)	Up to 100%	Activation gives cement-like properties (lab only)	High-energy milling boosts reactivity	[36]
Fine demolition recyclates (0–2 mm)	10–40%	Strength depends on parent concrete quality	Source sorting improves performance	[37]
Recycled concrete powder (RCP)	0–30%	20–25% acceptable; higher = strength drop	Comprehensive mechanical + microstructure testing	[38]
CDW and concrete powder	0–20%	10% replacement, with a 5% improvement	Can be used as 42.5 R Type II cement	This work

## Data Availability

The original contributions presented in this study are included in the article. Further inquiries can be directed to the corresponding author.
